# The Role of microRNA in Schizophrenia: A Scoping Review

**DOI:** 10.3390/ijms25147673

**Published:** 2024-07-12

**Authors:** Ke Li, Lin Zhu, Haibing Lv, Yulong Bai, Chuang Guo, Kuanjun He

**Affiliations:** College of Life Sciences and Food Engineering, Inner Mongolia Minzu University, Tongliao 028000, China; lk081821@163.com (K.L.); a2278250569@163.com (L.Z.); lv5421213@163.com (H.L.); 18147553467@163.com (Y.B.); guochuang@imun.edu.cn (C.G.)

**Keywords:** microRNA, schizophrenia, etiology, biomarker

## Abstract

Schizophrenia is a serious mental disease that is regulated by multiple genes and influenced by multiple factors. Due to the complexity of its etiology, the pathogenesis is still unclear. MicroRNAs belong to a class of small non-coding RNAs that are highly conserved in endogenous evolution and play critical roles in multiple biological pathways. In recent years, aberrant miRNA expression has been implicated in schizophrenia, with certain miRNAs emerging as potential diagnostic and prognostic biomarkers for this disorder. In this review, our objective is to investigate the differential expression of miRNAs in schizophrenia, elucidate their potential mechanisms of action, and assess their feasibility as biomarkers. The PubMed electronic database and Google Scholar were searched for the years 2003 to 2024. The study focused on schizophrenia and miRNA as the research topic, encompassing articles related to biomarkers, etiology, action mechanisms, and differentially expressed genes associated with schizophrenia and miRNA. A total of 1488 articles were retrieved, out of which 49 were included in this scope review. This study reviewed 49 articles and identified abnormal expression of miRNA in different tissues of both schizophrenia patients and healthy controls, suggesting its potential role in the pathogenesis and progression of schizophrenia. Notably, several specific miRNAs, including miR-34a, miR-130b, miR-193-3p, miR-675-3p, miR-1262, and miR-218-5p, may serve as promising biological markers for diagnosing schizophrenia. Furthermore, this study summarized potential mechanisms through which miRNAs may contribute to the development of schizophrenia. The studies within the field of miRNA’s role in schizophrenia encompass a broad spectrum of focus. Several selected studies have identified dysregulated miRNAs associated with schizophrenia across various tissues, thereby highlighting the potential utility of specific miRNAs as diagnostic biomarkers for this disorder. Various mechanisms underlying dysregulated miRNAs in schizophrenia have been explored; however, further investigations are needed to determine the exact mechanisms by which these dysregulated miRNAs contribute to the pathogenesis of this condition. The exploration of miRNA’s involvement in the etiology and identification of biomarkers for schizophrenia holds significant promise in informing future clinical trials and advancing our understanding in this area.

## 1. Introduction

Schizophrenia (SCZ) is a pervasive neuropsychiatric disorder characterized by a diverse range of mental symptoms, encompassing multifaceted manifestations of cognitive, emotional, and behavioral abnormalities, thereby rendering its trajectory intricate and heterogeneous [1,2]. As a chronic condition, it can have detrimental effects on individuals and impose significant costs on families, society, and healthcare systems [3,4]. The development and progression of SCZ are influenced by a complex interplay of genetic, environmental, and neurobiological factors [5,6]. Genetic factors contribute significantly to an individual’s susceptibility to developing SCZ, with specific gene variations increasing the risk of the disorder [7]. Environmental factors, such as prenatal stress, substance abuse, urban upbringing, and social adversity, can also have a profound impact on the onset and course of SCZ [8,9]. The onset of SCZ has prompted the formulation of numerous theories regarding neurobiological factors, encompassing abnormalities in brain structure and function, imbalances in neurotransmitters (such as dysregulation of dopamine), inflammation, and dysregulation of the immune system. These factors play pivotal roles in the pathogenesis of SCZ. Dopamine, a neurotransmitter closely associated with emotional regulation, motivation, and cognitive function, is found to be abnormally elevated in individuals with SCZ, which correlates with symptoms such as auditory hallucinations, delusions, and cognitive impairments [10]. Therefore, an imbalance in the dopamine system may constitute a key factor in the pathogenesis of SCZ. Recent studies have indicated that inflammatory responses significantly contribute to the development of SCZ [11]. Aberrant release of inflammatory factors may lead to neuronal damage and brain dysfunction, thereby exacerbating disease progression [12]. Furthermore, inflammatory responses may be associated with symptom severity in SCZ, necessitating regulation as a crucial strategy for treatment intervention against this disorder [13]. The immune system serves not only as a defense mechanism against pathogens but also exerts significant influence on brain function and mental health [14]. Abnormal activation of the immune system can result in inflammatory responses and autoimmune reactions affecting normal neuronal functioning [15]. Thus, immune system abnormalities might be closely linked to SCZ pathogenesis. Therefore, gaining a more profound comprehension of the involvement of the dopamine system, inflammation, and immune response not only enhances understanding but also provides valuable guidance for future preventive treatment strategies. Simultaneously, the existing diagnostic approaches for SCZ depend on subjective assessments, lacking objective measures to precisely evaluate the patient’s condition [16]. Culture also holds a crucial position in understanding the causes, diagnosis, and treatment of SCZ [17,18]. Introducing an objective diagnostic method would facilitate the development of more targeted treatment plans for effective patient management in the future.

**Figure 1:**
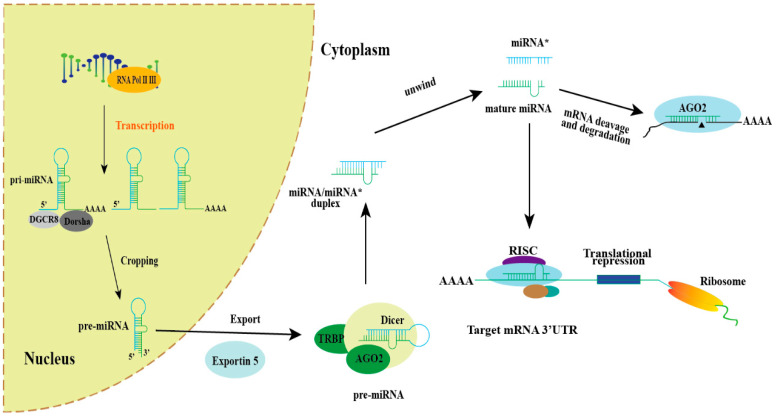
Schematic diagram of miRNA biogenesis and function. In general, the biogenetic pathway and related functions of miRNAs in cells begin with the transcription of RNA polymerase II/III, which is transcribed from genomic DNA into primary transcripts (pri-miRNAs). Pri-miRNA is cleaved by Dorsha/DGCR8 in the nucleus to produce miRNA precursors (pre-miRNA), which are exported to the cytoplasm via exportin-5 and further processed into double-stranded RNA double-stranded by Dicer/TRBP: miRNA*/miRNA. Mature miRNAs can associate with RNA-induced silencing complex (RISC) assemblies, thereby directing the translational repression of target mRNA, while the complementary strand, miRNA*, undergoes degradation.

MicroRNAs (miRNAs) constitute a class of diminutive non-coding RNAs, encoded by endogenous genes of approximately 18–25 nucleotides in length, which play crucial roles in development and disease [19]. The cellular biogenesis of miRNAs is a complex, multistep process that involves transcription from various genomic regions, including the exons and introns of host genes as well as intergenic fragments [20]. The biosynthesis of miRNAs begins in the nucleus, and mature miRNAs are produced in the cytoplasm. MiRNAs are predominantly transcribed via RNA polymerase II-dependent transcription, and then undergo capping, splicing, and polyadenylation to form primary miRNAs (pri-miRNAs), which have one or more hairpin structures and can be up to thousands of nucleotides in length [21,22]. The Drosha–DiGeorge Syndrome Critical Region 8 (DGCR8) complex mediates the processing of pri-miRNAs into precursor hairpins, which have an approximate length of 70 nucleotides. These precursor hairpins, known as pre-miRNAs, are then translocated into the cytoplasm via exportin-5 through the nuclear pore [23,24]. Another RNA enzyme, Dicer, breaks down pre-miRNAs into double-stranded RNA duplexes in the cytoplasm, i.e., miRNA strands and their complementary sequences (miRNA/miRNA*). MiRNAs orchestrate post-transcriptional gene silencing by precisely targeting the 3′ untranslated region (UTR) of mRNA, with particular emphasis on the seed region located at the 5′ end spanning nucleotides 2–7 [25]. Mature miRNAs can bind to the Ago-2-containing RNA-induced silencing complex (RISC) and mediate gene silencing, resulting in mRNA breakage or translational repression, while the other miRNA* strand is degraded [26,27]. As a major regulator of basic biological processes, miRNAs are able to regulate cell differentiation, proliferation, and apoptosis [28]. MiRNAs exert their biological functions by inhibiting downstream gene expression, mainly through transcriptional inhibition and mRNA cleavage or degradation, and indirectly regulate pathophysiological states [29]. MiRNAs bind to complementary target sequences in mRNA, interfere with translation mechanisms, alter or prevent the production of protein products, and thus regulate mRNA expression through different pathways [30]. The binding of miRNAs to target mRNAs also triggers the binding of mRNA attenuators, leading to mRNA instability, decreased expression levels, and even degradation [31,32,33]. MiRNAs have garnered extensive research attention due to their pivotal role in neuronal development and brain function [34]. Numerous studies have unequivocally demonstrated the active involvement of miRNAs in the pathophysiological mechanisms underlying SCZ [35,36,37]. The process of miRNA biogenesis is illustrated in Figure 1.

MiRNAs play major roles in brain development and function, regulation of neural development, neurotransmitter synthesis, and neural signaling [38,39,40,41]. Studies have shown that miRNAs are implicated in the pathophysiology and pathogenesis of many diseases [42,43,44,45,46]. This review provides a comprehensive overview of dysregulated miRNA expression patterns in individuals with SCZ, alongside recent advancements in the pathogenesis hypothesis involving miRNAs for this disorder.

## 2. Method

The present scope review was carried out following the PRISMA-ScR methodology (the preferred reporting item for extended meta-analysis of systematic Review and Scope evaluation) [47]. A comprehensive search of research articles published between 2003 and 2024 was conducted using both the PubMed database and the Google Scholar database. The most recent search was performed on 15 June 2024. At least two authors conducted a thorough screening of the title and abstract of each article to determine its eligibility for full-text review. Further evaluation was only conducted on studies that could not be definitively excluded based on the information provided in the title and abstract. Subsequently, two other authors assessed each remaining full-text article to ascertain its eligibility for inclusion in the study. This study has set up a file in the OSF database registration, registration DOI: https://doi.org/10.17605/OSF.IO/863RE.

Inclusion criteria:

- Articles written in English.
- Articles containing relevant keywords related to SCZ, miRNA, biomarkers, etiology, and mechanism of action.
- Articles with an abstract available

Exclusion criteria:

- Incomplete or unavailable full text.
- Articles written in languages other than English.
- Articles with weak relevance to the scope of this review.

## 3. Results

**Figure 2:**
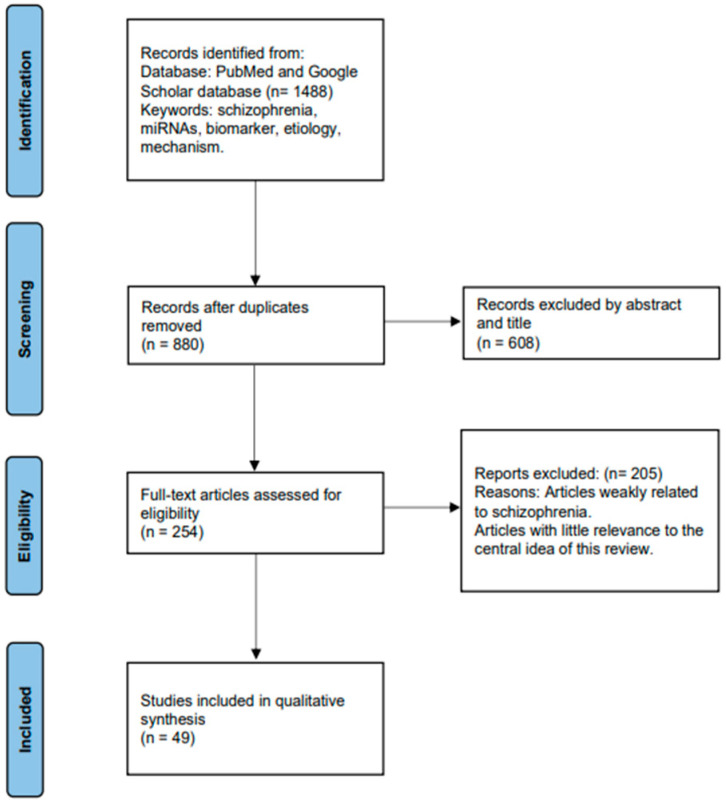
Process of data retrieval.

The studies included in this review all investigate the role of miRNAs in SCZ. Initially, a comprehensive search yielded a total of 1488 articles related to the association between SCZ and miRNAs. After removing duplicates, the remaining articles underwent screening based on their titles and abstracts. Subsequently, 254 articles were thoroughly analyzed in full text and selected for inclusion in this study. Among these 254 articles, 49 compared miRNA expression differences between SCZ patients and healthy controls (HCs), observing improvements in miRNA expression levels following medication. Additionally, significant differences in miRNA expression before and after treatment were found by some studies, suggesting their potential as biomarkers for SCZ. Furthermore, certain studies have elucidated the regulatory mechanisms through which miRNAs may contribute to the pathogenesis of SCZ. The process of article selection is illustrated in Figure 2 using the PRISMA Flowchart.

The review presents a comprehensive review of the role of miRNAs in SCZ. It details the differential expression of various miRNAs across tissues, including peripheral blood, prefrontal cortex (PFC), and temporal gyrus, in SCZ patients compared to HCs. MiRNAs exhibit a close association with SCZ, wherein their aberrant expression extensively contributes to the initiation and progression of the disorder, thereby potentially serving as crucial biomarkers for its diagnosis. The relevant pieces of research regarding miRNA dysregulation in SCZ can be seen in Table 1. Key findings include the upregulation of miR-195, miR-574-5p, miR-1827, miR-4429, miR-30e-3p, and miR-137 in peripheral blood [48,49,50,51], with miR-195 linked to cognitive impairment in female patients [52]. In monocytes from peripheral blood, miR-124-3p was upregulated [53], while miR-21-5p was downregulated [54,55]. Additionally, a two-fold increase in miR-206 expression was detected in extracellular vesicles from patient blood samples [56]. MiR-9 and miR-9-5p showed decreased expression levels in both peripheral blood and the PFC of SCZ patients [57,58]; however, miR-9-5p was upregulated in the superior temporal gyrus (STG) [57,58]. Conversely, the expression levels of miR-181b were up-regulated in PFC, STG, and the dorsolateral prefrontal cortex (DLPFC) [59,60]. These findings suggest that different patterns of miRNA upregulation or downregulation may influence neuronal differentiation and development processes in both peripheral blood and brain tissue, contributing to the pathogenesis of SCZ.

In recent years, miRNAs have emerged as promising diagnostic biomarkers for SCZ. For example, in two studies, miR-34a showed the most significant differential expression in mononuclear cells and the PFC of SCZ patients, suggesting its potential as a diagnostic marker for SCZ [61,62]. MiR-130b and miR-193a-3p were significantly elevated in patient plasma samples but were notably suppressed following antipsychotic treatment, indicating their potential as prognostic biomarkers for SCZ [63]. Additionally, abnormal expression of miR-675-3p, miR-1262, and miR-218-5p in peripheral blood accurately diagnosed treatment-resistant schizophrenia (TRS) patients [64,65], contributing to a more accurate diagnosis of SCZ. The identification of miRNAs as biomarkers offers promising avenues for early detection, monitoring of disease progression, and evaluation of treatment efficacy in SCZ. Modulating these miRNAs, either promoting or inhibiting their activity, can be achieved through targeted manipulation. This approach provides potential therapeutic benefits for treating psychiatric disorders.

Increasing evidence suggests that dysregulation of miRNAs may play a significant role in the etiology of SCZ by disrupting the mechanisms of neuronal development and plasticity, thereby leading to the onset of the disorder. Dopamine and glutamate are important in the pathophysiology of SCZ [66,67,68]. MiRNAs, as a new class of dopamine and glutamate modulators, can influence neurotransmission and neural signaling by modulating the synthesis and release of neurotransmitters, such as dopamine and glutamate, and thus influence the pathogenesis of SCZ [69]. In recent years, SCZ and the immune-inflammatory response have received close attention [70]. Abnormalities in immune inflammation are present in patients with SCZ [71], and miRNAs have been found to regulate the function of immune cells and the expression of inflammatory factors and thus influence the immune inflammatory response in studies of SCZ. The regulation of gene expression is critically influenced by miRNAs, which exert their effects on SCZ-related behaviors through targeting epigenetic processes, synaptic signaling, and cell–cell interactions within the brain.

**Table 1:** Differential expression of miRNAs in SCZ.

Species	Tissues	Study Population	Aberrantly Expressed miRNA(s)	Main Findings	Limitations	Future Advances	Potential Clinical Applications	References
Human	Peripheral blood	*n* = 118 patients with a first episode of SCZ, *n* = 47 HCs	miR-195 ↑	The expression level of miR-195 is significantly negatively correlated with the protein level of BDNF in patients. miR-195 may affect the cognitive function of SCZ patients by regulating the expression level of BDNF protein.	No vertical or intervention design has been conducted to determine the causal relationship between miR-195, BDNF mRNA and protein levels, and cognitive function. The relationship between peripheral BDNF levels and central nervous system levels in patients with SCZ remains to be elucidated.	The diagnosis and treatment of cognitive deficits may consider the miRNA–BDNF mechanism, as well as BDNF and other neural plasticity mechanisms.	The dysregulated expression of miRNAs in SCZ represents a promising therapeutic target for the development of effective treatment strategies.	[48]
	PBMC	*n* = 32 patients with SCZ, *n* = 48 HCs	miR-124-3p ↑	The upregulation of miR-124-3p is associated with the downregulation of BDNF, and antipsychotic drug treatment can modulate the expression levels of this network.	Small sample size. These findings are only available from PBMCs and should be compared with brain observations with caution. Patients were administered various classes of antipsychotic medications, potentially confounding the outcomes.	Future studies should control the types of drugs taken and conduct in-depth studies in brain tissue.	Regulating the balance of the miRNA–BDNF network may help restore the normal function of the BDNF signaling pathway, thereby improving the symptoms of SCZ.	[53]
	Exosome from blood	*n* = 49 patients with a first episode of SCZ, *n* = 46 HCs	miR-206 ↑	In the blood exosomes of SCZ patients, the expression level of miR-206 is significantly upregulated. MiR-206 can directly interact with the mRNA of BDNF, leading to the downregulation of BDNF expression, thereby exerting a negative impact on animal cognitive function.	Small sample size. A group of potential blood exosomal miRNAs were found but not validated as biomarkers for SCZ.	The functional role of miRNAs in psychopathology can be further explored, including their effects on neurodevelopment and neuroplasticity.	The abnormal expression of miRNA in SCZ may serve as a potential therapeutic target and relevant therapeutic strategies can be developed.	[56]
	Peripheral blood	*n* = 32 patients with SCZ, *n* = 48 HCs	miR-9-5p ↓	The down-regulation of miR-9-5p in the peripheral blood of patients with SCZ may promote the occurrence and development of SCZ	Limited sample size. Insufficient research on the specific function and mechanism of action of miRNAs.	To further investigate the function of SCZ-associated miRNAs and their interactions with other genes and signaling pathways.	The investigation into the mechanism underlying miRNA action holds great potential for unveiling novel therapeutic targets and facilitating the development of innovative drugs with clinical applicability.	[57]
	PFC	*n* = 13 patients with SCZ, *n* = 21 HCs	miR-9 ↓	MiR-9 is down-regulated in the PFC of SCZ patients and may play a key role in the pathogenesis of SCZ.	The sample size of the study is limited, and there is a lack of comprehensive investigation on miR-9.	To further explore the pathogenesis of miRNA in SCZ and clarify the complex relationship between miRNA dysregulation and the development of SCZ.	The identification of differentially expressed miRNAs in SCZ as biomarkers may provide valuable insights for clinical diagnosis and treatment.	[58]
	Neural progenitor cell	*n* = 6 patients with SCZ, *n* = 8 HCs	miR-9 ↓	Some SCZ patients exhibit abnormal regulation of miRNA-9 originating from neural precursor cells. Overexpression of miR-9 leads to the reduced migratory capacity of neural precursor cells.	The sample size was small and involved only patients with specific subtypes of SCZ.	The regulatory mechanism of miR-9 and its specific role in neurodevelopment and brain function can be further studied.	By modulating the expression of miR-9 to restore proper neuromigration and connectivity functions, miR-9 holds potential for diagnosing and predicting the onset and progression of SCZ.	[72]
	Plasma	*n* = 400 patients with SCZ, *n* = 213 HCs, and 162 patients with nonschizophrenia psychiatric disorders	miR-130b ↑, miR-193a-3p ↑	The expression levels of miR-130b and miR-193a-3p are significantly increased in SCZ, and their levels decrease significantly with symptom relief after treatment. The levels of circulating miRNAs may be associated with the onset and symptom relief of SCZ.	Although the study used two independent cohorts for verification, the sample size was still small. No endogenous reference miRNAs were available for the normalization of qRT-PCR data in this study. The calculated recovery rate of miR-16 is relatively low, potentially compromising the sensitivity of miRNA detection and impeding the capture of valuable signals.	Through in vitro and in vivo experiments, the specific roles of miR-130b and miR-193a-3p in the pathogenesis of SCZ and their effects on neurological function were explored.	It is further confirmed that the two miRNAs can be used as diagnostic markers for SCZ and may be used to develop clinical diagnostic tools.	[63]
	Plasma exosome	*n* = 9 patients with refractory SCZ, *n* = 50 patients with non-refractory SCZ, *n* = 59 HCs	miR-675-3p ↑	The alteration in miR-675-3p expression levels pre- and post-CLO treatment provides evidence for the significant involvement of miRNAs in the pathogenesis of treatment-resistant SCZ.	The sample size of microarray studies was small. The severity and medication history of patients in the study were different. The lack of optimal criteria in the study of exosomes may be a limitation of qPCR.	Long-term follow-up studies of patients can be conducted to explore the pathogenesis.	Individualized treatment options can be provided based on an individual’s genetic and epigenetic changes.	[64]
	PBMC	*n* = 82 patients with SCZ, *n* = 43 HCs	miR-21 ↑	The expression of miR-21-5p and miR-21 is upregulated in the PBMCs of patients with SCZ, and the expression level of miR-21 undergoes significant changes after antipsychotic drug treatment, which is associated with symptom improvement.	The sample size was small.	Future studies could predict the specific mechanisms of miR-21 downregulation. that improve symptoms.	MiR-21 may be a potential diagnostic marker as well as a potential therapeutic target.	[54]
	PBMC	*n* = 39 patients with SCZ, *n* = 50 HCs	miR-21-5p ↑	The expression level of miR-21-5p is upregulated in PBMCs from patients with SCZ.	The study’s relatively limited sample size may constrain the statistical power.	Future research endeavors could potentially enhance the sample size and further explore the underlying mechanism of miR-21-5p in SCZ.	MiR-21-5p may serve as a biomarker for the clinical diagnosis of SCZ.	[55]
	Whole blood	*n* = 40 patients with SCZ, *n* = 40 HCs	miR-574-5p ↑, miR-1827 ↑, miR-4429 ↑	MiR-574-5P, miR-1827, and miR-4429 may serve as potential biomarkers for SCZ. Through analysis of the miRNA–gene interaction networks, the regulatory relationships between these three miRNAs and genes related to SCZ have been identified.	The sample size was limited. The study used different data sets for analysis, and there may be some heterogeneity.	The biological function and potential clinical application of miRNA can be further investigated.	MiRNAs may serve as potential diagnostic markers for the diagnosis of SCZ as well as potential targets for new drugs.	[49]
	PFC	*n* = 21 patients with SCZ, *n* = 21 HCs	miR-181b ↑	The expression of miR-181b was significantly upregulated in the PFC of individuals with SCZ, suggesting its potential involvement in the pathogenesis of this disorder.	The sample size of the studies was limited and focused exclusively on the temporal cortex, without including other regions of the brain.	Future studies could cover more brain regions and miRNAs to fully understand the role of miRNAs in SCZ.	MiR-181b can be used as a biomarker of SCZ for early diagnosis and prediction of disease progression.	[59]
	STG and DLPFC	*n* = 36 patients with SCZ, *n* = 36 HCs	miR-181b ↑	The expression of miR-181b is significantly increased in STG and DLPFC of patients diagnosed with SCZ. Based on the prediction of target genes for miR-181b and pathway analysis, it is suggested that miR-181b may play a crucial role in human brain function and development.	The study had a limited sample size, and no subsequent investigation was conducted into the pathogenesis of SCZ.	The impact of miRNA on SCZ could be further investigated in additional brain tissues in future studies.	The potential application of miR-181b as a diagnostic marker in clinical settings holds promise for providing enhanced accuracy in the diagnosis of SCZ patients.	[60]
	Peripheral blood	*n* = 51 patients with SCZ, *n* = 51 HCs	miR-30e-3p ↑	Validated the differential expression of miR-30e-3p in SCZ and identified its candidate target genes. Further explored the potential biological functions and interaction networks of miR-30e-3p through functional enrichment analysis and PPI network analysis.	The targeting relationship between miRNA and target genes has not been further verified at the cellular level. The specificity of the potential biomarkers obtained in SCZ remains to be validated.	In future studies, the functional role of miRNA and mRNA can be verified through cell or animal models to reveal the pathogenesis.	In the clinic, miR-30e-3p may serve as a potential diagnostic marker for SCZ diagnosis.	[50]
	Whole blood	*n* = 215 patients with SCZ (104 EOS and 111 AOS), *n* = 72 unaffected first-degree relatives, *n* = 31 patients with bipolar disorder, n = 100 HCs.	miR-137 ↑	In EOS and AOS patients, there are differences in the expression levels of miR-137, which plays a significant role in the regulatory network of SCZ and may serve as a biomarker for the aberrant expression of neurodevelopment-related miRNAs.	Abnormal miRNA expression has been observed in peripheral blood, and whether these differences persist in brain or neural tissue remains to be confirmed. The expression of free miRNA in whole blood was detected, revealing distinct miRNA profiles specific to different blood cell subtypes, which may exhibit variations in proportions across study groups.	Future studies could delve deeper into whether miRNAs are abnormal in the brain or neural tissue and understanding the underlying mechanisms of genetic susceptibility.	The level of miR-137 expression may contribute to the early diagnosis and prediction of psychiatric disorders, which can help doctors more accurately determine the type of disease and prognosis of patients.	[51]
Mice	Hippocampus		miR-146a-5p ↑, miR-200b-3p ↑	MiR-146a and miR-200b modulate cognitive function by targeting NMDA receptor subunits, and their upregulation leads to cognitive impairment.	Only male rats were used in the study. Behavioral tests were not conducted using more emotional and cognitive-behavioral approaches.	This study can be linked to human studies and further study the regulation of NMDA receptors by miR-146a and miR-200b.	Based on the important role of miR-146a and miR-200b in cognitive function, the potential clinical application of these miRNAs as therapeutic targets is further explored to improve the therapeutic effect of cognitive dysfunction.	[73]
	Hippocampus		miR-138-5p ↑, miR-184 ↑, miR-21-3p ↑, miR-340-5p ↑, miR-542-3p ↑, miR-6324 ↑, miR-455-3p ↓, miR-483-3p ↓	The administration of MK-801 and CLO significantly modulated the expression profile of miRNAs in the hippocampus of rats.	The experimental design was not sufficiently rigorous, and the miRNA analysis did not encompass the CLO treatment group. Lack of other behavioral tests associated with SCZ. The investigation of miRNA expression changes was limited to the hippocampal region, with no examination conducted in other cerebral areas.	The future focus should primarily revolve around investigating the functional implications of significantly dysregulated miRNAs and their target genes in SCZ, while also validating the potential diagnostic and therapeutic utility of rno-miR-184.	MiR-184 may be a potential therapeutic target for SCZ. The study also found that CLO may play a role in the regulation of estrogen signaling pathways, and CLO may be used as an adjunct to antipsychotic therapy.	[74]

Note: ↓ or ↑: MiRNA expression levels were up-regulated or down-regulated. Abbreviations: SCZ, Schizophrenia; MiRNAs, microRNAs; HCs, healthy controls; BDNF, brain-derived neurotrophic factor; PBMC, peripheral blood mononuclear cell; PFC, prefrontal cortex; STG, superior temporal gyrus; EOS, early-onset schizophrenia; AOS, adult-onset schizophrenia; DLPFC, dorsolateral prefrontal cortex; NMDA, N-methyl D-aspartate; CLO, clozapine; qPCR, quantitative real-time PCR; PPI, producer price index.

## 4. Discussion

MiRNAs can modulate the expression of specific genes through the RNA interference pathway, thereby affecting the function and development of various glial cells in the brain [75]. The brain and blood of patients with SCZ exhibit differential miRNA expression compared to NCs [76,77], suggesting that these variations may underlie the pathogenesis of SCZ, and the targets have the potential to serve as therapeutic targets for antipsychotic drugs.

The expression level of miR-195 in peripheral blood was significantly elevated in patients diagnosed with SCZ [48]; however, there were also studies that reported no significant difference [76]. High expression of miR-195 is closely associated with decreased levels of brain-derived neurotrophic factor (BDNF) protein, and miR-195 can specifically target the 3′UTR of BDNF, thereby leading to alterations in cognitive function [60]. Studies on cognitive impairment have revealed an association between miR-195 and cognitive deficits in SCZ patients, particularly among female individuals [52]. Therefore, it is plausible that miR-195 may be involved in the underlying mechanism of gender-related cognitive deficits in SCZ. The expression of miR-124-3p was upregulated in the peripheral blood mononuclear cells (PBMCs) of patients with SCZ, while the expression of miR-206 and miR-132-3p did not show significant differences [53]. Following 12 weeks of continuous administration of antipsychotics, BDNF expression was upregulated, whereas all three miRNAs were downregulated; furthermore, a positive correlation was observed between BDNF and all three miRNAs [53]. In another study, the expression of miR-206 in blood exosomes from SCZ patients tripled [56]. The dependent regulation of SCZ protective alleles on 5′-nucleotidase, cytoplasmic II (NT5C2) may lead to the promotion of SCZ by miR-206 [78]. In animal experiments using a neurodevelopmental model of SCZ mice with N-methyl-D-aspartate receptor (NMDAR) dysfunction and treated with ketamine, the expression of miR-132-3p significantly increased in the PFC of female mice but showed no significant change in male mice, indicating sexual dimorphism [79]. Future studies could further explore whether similar sex differences exist for miR-132-3p in humans. Pri-miR-9-2 is a neurogenic precursor that primarily regulates the production of miR-9. Studies have indicated that the expression of miR-9 and miR-9-5p in the peripheral blood and PFC of patients with SCZ is down-regulated, while the expression of miR-9-5p is up-regulated in the STG. This suggests that miR-9 may be involved in the etiology of SCZ [57,58]. Another study discovered that miR-9-5p is down-regulated in peripheral blood samples from patients experiencing first-episode SCZ, and its target genes are involved in the biological pathways related to neuronal differentiation and nervous system development [80]. Additionally, there exists a strong correlation between miR-9-5p and miR-137 [81], which has been proven as a potential diagnostic biomarker for SCZ [51,82,83]. The expression of miR-21-5p in the PBMCs of SCZ patients was downregulated [54,55], and the expression of miR-21 significantly decreased after treatment with antipsychotic drugs [54]. Studies have indicated that there are miRNA binding site variants (MBSVs) present in the genes targeted by miR-21-5p [84], which may be associated with SCZ. Additionally, miR-574-5p, miR-1827, and miR-4429 were upregulated in the blood of patients with SCZ, showing predictive and diagnostic value [49]. Multiple studies have demonstrated that the expression of miR-181b is upregulated in the PFC and STG regions among SCZ patients, while its expression is also significantly altered in PBMCs [55,59,60,85]. These findings contribute to a deeper understanding of the role played by miRNAs in the pathogenesis of SCZ. The abnormal expression of miRNA in patients with SCZ is associated with the regulation of receptors and genes in the nervous system. The up-regulation of miR-30e-3p has been observed in peripheral blood samples from SCZ patients [50]. NMDAR dysfunction plays a role in the pathophysiology of SCZ, which may be influenced by miRNA-mediated regulation [86]. In rat models with down-regulated NMDAR subunits, miR-146a-5p and miR-200b-3p were found to be up-regulated [73]. Whole blood tests revealed elevated levels of miR-137 expression in both early-onset schizophrenia (EOS) and adult SCZ (AOS) patients [51]. Furthermore, miR-137 expression may be linked to underlying mechanisms contributing to genetic susceptibility for SCZ [87,88,89,90,91]. Using SCZ experimental animal models, it was found that olanzapine treatment of SCZ mice resulted in the downregulation of miR-223 expression in neuronal granules and neuronal exosomes [92]. Previous studies have shown that miR-223 is enriched in mouse astrocytes and secreted by exosomes, and this enrichment may contribute to the occurrence of SCZ [37]. After treating an MK-801-induced rat SCZ model with clozapine (CLO), hippocampal analysis showed significant differences in the expression of 14 miRNAs between the MK-801 + CLO group and the MK-801 group. Eight miRNAs were differentially expressed in the MK-801 group, including six upregulated (miR-138-5p, miR-184, miR-21-3p, miR-340-5p, miR-542-3p, and miR-6324) and two downregulated (miR-455-3p and miR-483-3p). In particular, after MK-801 treatment there was an upregulation of miR-184 followed by a downregulation after MK801 + CLO treatment [74]. Further research is needed to determine whether miR-184 can be used as a diagnostic marker for SCZ. The abnormal expression of miRNAs in peripheral blood and brain has an impact on SCZ, and it can be concluded that miRNAs have important theoretical, experimental, and clinical significance for SCZ. MiRNAs as a biological diagnostic marker for SCZ are still being explored, and although the mechanism of action is still unclear, miRNAs as diagnostic markers for SCZ have great potential.

Several studies have demonstrated the utility of miRNAs as biomarkers for the diagnosis of SCZ. The most significant difference in miR-34a expression was observed in the monocytes [61] and PFC [62] of SCZ patients, suggesting its potential as a diagnostic marker for SCZ. In plasma samples from SCZ patients, there were significantly elevated levels of miR-130b and miR-193a-3p [63]. After one year of treatment with aripiprazole and risperidone, the expression levels of miR-130b and miR-193a-3p were significantly suppressed, suggesting their potential as prognostic biomarkers for SCZ [63]. The changes of plasma exosomal miRNA in patients with TRS are closely related to neuronal function [64]. miR-675-3p [64], miR-1262, and miR-218-5-p [65] have been found, in the peripheral blood of TRS patients, to have potential as diagnostic markers for TRS, contributing to a more accurate diagnosis of SCZ. Studies related to miRNAs as biomarkers of SCZ are still in the exploratory stage, and more research is needed to support the relationship between the two. In the future, it is possible to treat SCZ by modulating the expression of specific miRNAs, which may become a new therapeutic strategy for the treatment of SCZ. Research on miRNAs as biomarkers of SCZ can also help to reveal the pathogenesis of psychiatric disorders.

MiRNAs are involved in the regulation of gene expression and play a crucial role in brain development and neuroplasticity [93]. The role of miRNA in the pathogenesis of SCZ is still being explored, but the significance of miRNA in the pathogenesis of SCZ should not be disregarded. The expression levels of certain miRNAs in the blood of patients with SCZ are significantly different compared with those of normal people, and these abnormally expressed miRNAs may be involved in the onset and development of SCZ. Patients with SCZ have abnormal neurodevelopment and plasticity, which in turn leads to brain dysfunction. MiRNAs affect neural development and plasticity and play a key role in the regulation of brain neural development and function [94], which suggests that the two are closely related. Abnormal expression of miRNAs in SCZ patients may disrupt neural structural functions, such as normal neuronal function, dendritic development, and synaptic connectivity, thus affecting neural network construction in the brain [39,95]. Some studies have now identified potential mechanisms of miRNA action in SCZ. Dopamine and glutamate are important in the pathophysiology of SCZ [66,67,68]. MiRNAs, as a new class of dopamine and glutamate modulators, can influence neurotransmission and neural signaling by modulating the synthesis and release of neurotransmitters, such as dopamine and glutamate, and thus influence the pathogenesis of SCZ [69]. In recent years, SCZ and the immune-inflammatory response have received close attention [70]. Abnormalities in immune inflammation are present in patients with SCZ [71], and miRNAs have been found to regulate the function of immune cells and the expression of inflammatory factors and thus influence the immune inflammatory response in studies of SCZ. Studies have shown that increased neuroinflammation is associated with the pathogenesis of SCZ, with increased oxidative stress and inflammation in blood and brain tissue in SCZ patients [96,97].

The regulation of gene expression is critically influenced by miRNAs, which can modulate behaviors related to SCZ through their targeting of epigenetic processes, synaptic signaling, and cell–cell interactions within the brain. Consequently, the investigation of miRNAs’ involvement in neurogenesis and pathogenesis of neurological disorders has garnered significant attention [98]. Elucidating and revealing the neuronal mechanism of miRNA has been the key to research. Finding a potential mechanism to treat psychiatric disorders could guide the development of targeted drugs that target specific sites of action to improve patients’ symptoms.

This review has several limitations. The included studies show variability in patient demographics, disease severity, and treatment status, all of which could affect miRNA expression levels. Additionally, many studies have small sample sizes, which reduces their statistical power and limits the generalizability of their findings. Despite these limitations, a consistent trend of specific dysregulated miRNAs in individuals with SCZ was identified. Most studies are cross-sectional and do not provide longitudinal data, making it difficult to track changes in miRNA expression over time or to understand their relationship to disease progression and treatment response. Future research should focus on increasing sample sizes and employing longitudinal designs to better understand the dynamic changes in dysregulated miRNAs throughout the course of SCZ. In this scoping review, the evidence sources were not critically assessed; thus, no data regarding the critical evaluation of evidence sources was provided.

## 5. Conclusions

The dysregulation of miRNAs in SCZ has been extensively investigated, encompassing their potential roles as well as the diverse mechanisms underlying their dysregulation. We conducted a comprehensive search of the PubMed database and the Google Scholar database and ultimately identified 49 studies that met our predetermined criteria. Through meticulous sorting and analysis of these papers, we have reached the conclusion that differentially expressed miRNAs play a pivotal role in promoting the onset and progression of SCZ. Furthermore, our findings suggest that miRNAs may be involved in the pathogenesis of SCZ through regulatory mechanisms that involve dopamine, glutamate, and the immunoinflammatory response. These conclusions provide cutting-edge evidence regarding the role and potential mechanisms underlying miRNA involvement in SCZ. The findings from all the selected studies indicate the significance of miRNAs in SCZ. However, it is important to note that, due to variations in the tissue samples utilized across studies, there exists variability in evaluating the impact of miRNA on SCZ. Further elucidation of their regulatory mechanisms and involvement in SCZ can shed light on the disease’s pathophysiological features, offering insights into its diagnosis, treatment, and prevention. Modulating miRNA expression or activity holds promise for restoring normal gene expression and neurobiological functions to ameliorate SCZ symptoms, thus paving the way for improved treatments and management strategies.

## Abbreviations

SCZ	schizophrenia
MiRNAs	microRNAs
PRISMA-ScR	Preferred Reporting Items for Systematic reviews and Meta-Analyses extension for Scoping Reviews
Pri-miRNAs	primary miRNAs
DGCR8	DiGeorge Syndrome Critical Region 8
UTR	untranslated region
RISC	RNA-induced silencing complex
HCs	healthy controls
BDNF	brain-derived neurotrophic factor
PBMC	peripheral blood mononuclear cell
NT5C2	5′-nucleotidase, cytoplasmic II
NMDAR	N-methyl-D-aspartate receptor
NMDA	N-methyl-D-aspartate
PFC	prefrontal cortex
RT-qPCR	real-time PCR
MBSVs	miRNA-binding site variants
STG	superior temporal gyrus
SNPs	single nucleotide polymorphisms
EOS	early-onset schizophrenia
AOS	adult-onset schizophrenia
DLPFC	dorsolateral prefrontal cortex
CLO	clozapine
qPCR	quantitative real-time PCR
PPI	producer price index
TRS	treatment-resistant schizophrenia
